# Comparing Hepatitis C Virus Screening in Clinics Versus the Emergency Department

**DOI:** 10.5811/westjem.2021.11.53870

**Published:** 2022-03-17

**Authors:** Rebecca Hluhanich, James S. Ford, Devin Bruce, Tasleem Chechi, Stephanie Voong, Souvik Sarkar, Patricia Poole, Nam Tran, Larissa May

**Affiliations:** * UC Davis Health, Hepatology and Infectious Diseases Clinical Pharmacy, Sacramento, California; † UC Davis Health, Department of Emergency Medicine, Sacramento, California; ‡ UC Davis Health, Department of Internal Medicine, Division of Gastroenterology, Sacramento, California; § UC Davis Health, Department of Pharmacy, Sacramento, California; ¶ UC Davis Health, Department of Pathology and Laboratory Medicine, Sacramento, California

## Abstract

**Introduction:**

New evidence suggests that emergency department (ED)-based infectious diseases screening programs have utility. We aimed to compare clinic-based and ED-based hepatitis C virus (HCV) screening programs within a single health system, to identify key differences in HCV antibody (Ab) positivity and chronic HCV, as well as population demographics.

**Methods:**

In the clinic-based program, adults in the birth cohort (born 1945–1965) were screened for HCV. In the ED-based program, non-targeted HCV screening of all adults was conducted. We included patients screened between June 2019–June 2020. Patients were screened for anti-HCV Ab, and positive results were followed by HCV viral load (VL) testing. Our primary outcomes were seroprevalence of HCV Ab and HCV VL.

**Results:**

There were 1,296 and 12,778 patients screened for HCV in the clinics and the ED, respectively. In the clinic setting, 13 patients (1%) screened positive for HCV Ab and nine (69%) completed VL testing, which was positive in one patient (11%). In the ED, 1,053 patients (8%) screened positive for HCV Ab and 847 (80%) underwent reflex VL testing, which was positive in 381 patients (45%). In an ED birth cohort sub-analysis, Hepatitis C virus Ab seroprevalence was 15% (675/4521).

**Conclusion:**

In this study of patients in a single healthcare system, ED-based HCV screening was higher yield than clinic-based screening.

## INTRODUCTION

There are an estimated 150 million people with chronic hepatitis C virus (HCV) globally.^1^ In the United States (US), HCV is the most common bloodborne infection, and it is responsible for more deaths than any other chronic infectious disease in the country, largely because of its association with cirrhosis and hepatocellular carcinoma.^2^,^3^ For reasons that are unclear, HCV-related mortality has increased in recent decades, while mortality rates for 60 other notable infectious diseases, including human immunodeficiency virus (HIV) and hepatitis B virus (HBV), have decreased.^2^ In 2020 the US Centers for Disease Control and Prevention (CDC) released guidelines recommending HCV screening in all adults 18 years and older.^4^ With curative treatments becoming more accessible and affordable, systematic approaches to identifying infected individuals could drastically reduce the burden of disease.^5^,^6^

While screening for infectious diseases has historically been viewed as a primary care service, a growing body of evidence has emerged suggesting that emergency department (ED)-based screening protocols have utility, given the tendency for EDs to care for medically underserved and behaviorally high-risk populations.^7^,^8^ Screening programs based in EDs have demonstrated success in screening for infectious diseases including HCV, HIV, and HBV.^7^,^9^–^14^ However, using the ED as a setting for delivery of public health interventions remains controversial.^15^ There is limited data comparing HCV screening practices between ambulatory and ED settings; therefore, it remains unclear whether ED-based HCV screening programs provide utility, relative to traditional clinic-based programs.

Our health system implemented an ED-based HCV screening program in November 2018 and a clinic-based program in May 2019. In this study our goal was to compare clinic-based and ED-based HCV screening programs within a single health system to identify key differences in HCV antibody (Ab) positivity and chronic HCV, as well as population demographics and risk profiles.

## METHODS

### Overview

This was a retrospective cohort analysis comparing HCV screening initiatives between the ED and clinic setting within the University of California Davis Health system. On November 27, 2018, the health system implemented a non-targeted, ED-based HCV screening program of all adults undergoing phlebotomy as part of their ED workup. On May 7, 2019, our institution implemented an HCV screening program in its ambulatory care clinic (ACC) network for all individuals born between 1945–1965. The ACC-based screening program was pharmacist-driven, and we characterize the implementation of this program. The study institution’s ED-based HCV screening program has been previously described.^16^ These HCV screening programs are the result of a collaboration between the ED, primary care clinics, specialty pharmacy, the Division of Gastroenterology and Hepatology, the Sacramento County Health Department, and the local Federally Qualified Health Centers (FQHC). All authors had access to the study data and reviewed and approved the final manuscript. This study was determined not to be human subjects research by the UC Davis Institutional Review Board Quality Improvement Self-Certification Tool.

### Study Setting and Population

UC Davis Health is a quaternary referral, academic health system in northern California. The study ED is a Level I adult and pediatric trauma center that services a mixed urban and rural population and has more than 80,000 patient visits annually. Six primary care clinics from the ACC network were included in the study.

### Program Implementation

#### Pharmacist-Driven HCV Screening Program Design

Hepatology and infectious diseases clinical pharmacists collaborated with primary care clinics to conduct HCV screening. Education on HCV screening and management was provided to all practicing physicians at each site, and they were given the opportunity to opt into the pharmacist screening program. The screening initiative was implemented in six primary care clinics out of a total of 13 internal medicine primary care clinics in our health system. A total of 41 staff physicians and 58 medical residents opted into the program. The screening program consisted of a pharmacist and a patient navigator and used a report that identified patients who had an overdue Best Practice Advisory (BPA) for HCV screening based on birth years 1945–1965. Individual patient health records were reviewed for documentation of previous HCV Ab testing in outside records accessible via the Epic Care Everywhere interoperability platform (Epic Systems Corporation, Verona, WI) and were excluded if a result was present.

**Population Health Research Capsule**

What do we already know about this issue?

*Hepatitis C virus (HCV) screening is typically performed in clinic, but new data suggest that emergency departments (ED) are an important setting for screening.*

What was the research question?

*Is HCV antibody prevalence different between ED and clinic populations within the same health system?*

What was the major finding of the study?

*HCV antibody prevalence was 8-fold higher in the ED (8%) compared to the clinics (1%).*

How does this improve population health?

*Screening for HCV in the ED is high yield and can complement traditional clinic-based screening.*

Eligible patients were then notified by either Epic Mychart electronic messaging or a written letter 30 days prior to ordering HCV Ab testing. A phone number was provided to allow for patients to opt out of HCV screening. After the 30-day waiting period, pharmacists placed the HCV Ab order, which remained active for one year. For positive HCV Ab results, pharmacists reviewed results with patients by phone and placed orders for the HCV viral load (VL) and HCV genotype. Patients with negative HCV Ab tests were notified by either Mychart or written letter. The pharmacist notified physicians of positive HCV VL results and discussed the plan to disclose new diagnoses to patients, as well as to refer patients to hepatology clinic for treatment. If HCV VL testing was negative, the pharmacist discussed results with the patient via phone.

#### Brief Summary of ED Screening Program Design

All ED patients ≥18 years and born after 1945, who were having blood drawn for any clinical purpose and did not have a positive HCV RNA test result in the electronic health record (EHR) within the prior six months, were eligible for opt-out HCV screening. Upon entering any laboratory order into the EHR, a BPA notified the emergency clinician that the patient met screening criteria; at that point, clinicians were required to respond to continue with the order entry. Complete details of the ED-based program have been previously described.^16^

#### Hepatitis C Virus Laboratory Testing Protocol

HCV screening was conducted by testing blood samples with a chemiluminescent immunoassay that detects HCV Ab (Architect i1000, Abbott Laboratories, Abbott Park, IL). Positive HCV-Ab tests underwent diagnostic confirmation by measuring HCV RNA viral load (Cobas AmpliPrep/TaqMan, Roche Diagnostics, Basel, Switzerland). Results of HCV-Ab testing were typically available within 1–3 days. The results of VL testing were typically available within four days.

### Study Design

We performed a retrospective cohort analysis comparing outcomes between HCV screening settings (ACC vs ED), over a 12-month study period. We consecutively included all patients who received HCV testing as part of the ACC or ED screening programs, between June 6, 2019–June 5, 2020. We excluded from our analysis patients who were tested for HCV by emergency clinicians (unprompted by BPA). Data were abstracted directly from the EHR using computer-generated reports; ancillary research staff who procured the reports were not involved with the study and were blinded to the study aims and hypotheses. No manual chart review was performed. The ED data abstracted included age, gender, ethnicity/race, ED visit date, ED chief complaint, clinic HCV testing date, insurance type (Medicare, Medicaid/other public, self-insured/uninsured) and results of HCV testing. Data were stored in de-identified datasets, and each patient was given a unique identifier to maintain patient confidentiality. To prevent duplicate data, only a patient’s first ED visit where they received HCV testing was included in our analysis.

### Outcomes

The primary outcomes were HCV-Ab seropositivity (number positive/number tested) and the number of confirmed chronic HCV cases (defined as detectable HCV RNA viral load).

### Analysis

Data were described with simple descriptive statistics. Categorical variables were expressed as percentages and proportions, and continuous variables were expressed as means or medians (Q1–Q3). We provided 95% confidence intervals (CI) where appropriate. Comparisons between groups were made using Fischer’s exact test. We performed statistical analysis using Stata 15.1 (StataCorp LLC, College Station, TX).

## RESULTS

### Characteristics of Study Subjects

A total of 1,296 patients were screened in the ACC, and a total of 12,778 patients were screened in the ED during the study period. In the ACC, 3,569 patients were notified of their eligibility for HCV screening; 52 (2%) patients opted out, 1,296 (36%) patients completed screening, and 2,221 (62%) of patients did not complete screening within the study timeframe. The ED-based BPA was accepted by clinicians in 47% (12,778/27,270) of patient visits in which it fired. Patients screened in the ED were younger than those screened in outpatient clinics (mean age: 46 ± 16 years vs 66 ± 6 years). Gender data was similar between study cohorts. Most patients screened in the ACC were White, whereas race was more evenly distributed among patients screened in the ED (White: ACC = 72%; ED = 43%). Full patient characteristics are described in Table 1.

### Screening Results

HCV-Ab screening was reactive in 1% (13/1296, 95% CI: 0.4, 1.6) of patients screened in the ACC, compared to 8% (1,053/12,778, 95% CI: 7.8, 8.8) of patients screened in the ED. Follow-up VL testing was performed in 69% (9/13) of HCV-Ab reactive patients in the ACC, and in 80% (847/1,053) of HCV-Ab reactive patients in the ED. Viral load was positive in 11% (1/9, 95% CI: 0.2, 48.2) of patients tested in the ACC and 45% (381/847, 95% CI: 42, 48) of patients tested in the ED. The HCV-Ab seropositivity of ED patients tested in the birth cohort age 55–74 was 15-fold higher compared to those tested in the ACC (Table 2).

Most patients who screened positive for HCV Ab in the ED were in the birth cohort 55–74 years: 675/1,053 (64%); however, VL was more likely to be reactive in patients 18–54 years compared to those a 55–74 years old (50% [155/308, 95% CI: 45, 56] vs 42% [226/539, 95% CI: 38, 46), *P* = 0.02]. In the ED, no patients over 75 screened positive for HBV Ab (N = 14).

## DISCUSSION

Screening for HCV in the ED was higher yield than clinic-based screening. Disparities in HCV seropositivity suggest that ED-based infectious disease screening programs can complement traditional outpatient screening programs.

An estimated 2.4 million people in the United States are living with hepatitis C, and it is estimated that only half are aware of their HCV-positive status.^3^,^17^ Risk of contracting HCV has been shown to be especially high in individuals who are undomiciled, engage in high-risk sexual practices, share needles and other drug injection equipment, or have been incarcerated.^18^ These individuals also have lower rates of health insurance coverage and often have limited access to primary care services, contributing to frequent visits to the ED.^18^ In this study we found that patients who were tested for HCV in the ED were more likely to have had previous exposure to HCV (detectable HCV Ab) and were more likely to have chronic HCV (detectable HCV VL) than those who were tested as part of clinic-based initiatives.

The ED and ACC cohorts differed substantively in many key domains. These differences can be partially ascribed to the patient populations who were selected for screening. It is unsurprising that patients tested in the ACC setting were older, given that the ACC conducted birth cohort screening of patients born between 1945–1965, whereas the ED conducted non-targeted screening of adults. Historically, the birth-cohort age group has been classified as the highest risk population, due to iatrogenic exposures such as blood transfusions pre-1992 and dialysis, as well as lifestyle factors such as injection drug use.^19^ However, recent data has caused experts to question these risk profiles, leading the US CDC to extend its screening recommendations to all adults ≥ 18 years.^4^

The seroprevalence of HCV Ab in the ACC (1%) was similar to the national average of patients within a similar birth cohort (1.6%).^20^ The overall seroprevalence of HCV Ab in the entire ED cohort was 8%, which is similar to that reported by other ED-based, non-targeted HCV screening studies (6–13.2%).^13^,^21^–^23^ Interestingly, the seroprevalence of HCV Ab in the ED birth cohort (15%) was higher than what has been reported previously by other ED-based, birth cohort, HCV screening studies (6.3–9.9%).^12^,^22^,^24^ In our study, birth-cohort patients tested in the ED were 15-fold more likely to have had exposure to HCV (HCV-Ab seropositive), and nearly fourfold more likely to have active hepatitis C (HCV-VL seropositive), when compared to patients tested in the ACC. Patients tested in the ED were more likely to be non-White or of mixed ancestry, undomiciled, and uninsured/self-pay. This data implies a stark socioeconomic and demographic divide between ED and ACC patients, suggesting that lifestyle risk factors may be driving disparities in health outcomes. Future ED-based, HCV risk-factor studies could assist in identifying high-risk patients.

As the public health needs of communities continue to outpace the capacity of clinicians and public health authorities, the role of the pharmacist in leading screening initiatives has expanded to meet these needs. Pharmacist-driven public health initiatives have demonstrated success in myriad settings.^25^–^31^ In our study an outpatient HCV testing program led by a single pharmacist and program navigator was able to screen over 1,200 patients for HCV in one year. While the ACC screening program diagnosed only one new case of HCV, this modest result is likely attributable to the fact that screening was limited to the birth cohort; one would expect a higher yield of new HCV diagnoses if a universal screening protocol of all adult patients was adopted.

## LIMITATIONS

The results of our study must be interpreted in light of its limitations. This was a study from a single health system; so our findings may not be generalizable to all settings. The ED and ACC HCV screening programs differed in many ways. While the ACC employed a birth-cohort screening protocol, the ED universally screened all adults, which logically led to substantive differences in study populations. However, to account for this difference, we included a sub-analysis that compared only patients screened in the birth cohort in the ED to those screened in the ACC, which demonstrated an even more profound difference in HCV-Ab reactivity in the ED population. While the ED used an automated BPA that was integrated into the EHR, which would automatically initiate HCV orders (with clinician approval) on any patient undergoing phlebotomy, orders for HCV screening had to be manually entered by study pharmacists. While automated HCV test orders were accepted in only 47% of patients in the ED, this was still numerically greater than the 36% of eligible patients who completed testing in the ACC.

There may be several reasons why HCV testing uptake was low in the ACC. In the ACC, patients had one year from the time the HCV order was placed to go to the laboratory to complete testing; however, if they had no other reason to access laboratory services during that time, they were unlikely to receive HCV testing. Additionally, patients who may have had their testing done after the end date of the study period (June 5, 2020), would not have had this testing counted as part of this study. Since the ED cares for substantially more patients annually than the ACC, the ED cohort was much larger than the ACC cohort. Limited data were available with respect to the patient characteristics of study cohorts; future studies should further explore important population-level differences between testing settings, such as socioeconomic status, history of HIV, history of illicit drug use, and other potential risk factors for HCV infection. Linkage-to-care data were not available for this study. Since only a patient’s first HCV testing encounter was included in our analysis, the effect of frequent ED visits (and frequent HCV testing) from high utilizing individuals was not accounted for, which may have led to an overestimation of true ED testing yields.

## CONCLUSION

Emergency department-based screening for hepatitis C virus was higher yield than clinic-based screening. Disparities in HCV seropositivity highlight key demographic differences between settings and marked risk differences between these populations. Overall, these results contest the long-held dogma that infectious disease screening should be conducted only in the outpatient setting. Emergency department-based screening strategies complement traditional clinic-based screening strategies and may help provide these services to populations that otherwise would not be able to access them.

## Figures and Tables

**Table 1:** Patient characteristics stratified by screening setting

Characteristic	ED (N = 12,778)	ACC (N = 1296)	Difference (95% CI)
Age (years)1	46 ± 16	66 ± 6	20 (19, 21)
Female Gender	51% (6,502/12,776)	52% (674/1,296)	1 (−2 to 4)
Race			
White	43% (5,391/12,589)	72% (902/1,256)	29 (26, 32)
Black	18% (2,332/12,589)	8% (99/1,256)	11 (9, 12)
Asian	8% (970/12,589)	10% (121/1,256)	2 (0.3, 3.7)
Other/Mixed	31% (3,896/12,589)	10% (124/1,256)	21 (19, 23)
Undomiciled	8% (984/12,115)	<1% (1/1,296)	8 (7, 9)
Insurance Type			
Private	59% (7,587/12,778)	60% (781/1,296)	1 (−2 to 4)
Medicare	18% (2,324/12,778)	39% (506/1,296)	21 (18, 24)
Medicaid/Other Public	17% (2,216/12,778)	1% (9/1,296)	16 (15, 17)
Self/Uninsured	5% (651/12,778)	0% (0/1,296)	5 (5, 5)

1 Only patients between the ages of 55–74 years were eligible for screening in the ACC, whereas all patients ≥18 years in the ED were eligible for screening.

*ACC*, acute care clinics; *ED*, emergency department; *CI*, confidence interval.

**Table 2:** Hepatitis C virus results stratified by screening setting and age group.

	ED^1^		ACC
		
	Age 18–54	Age 55–74	Age 55–74
HCV-Ab Reactive	378/8243 (5%)	675/4521 (15%)	13/1296 (1%)
HCV-VL Positive	155/308 (50%)	226/539 (42%)	1/9 (11%)

1 No patients over 75 years tested positive for HCV Ab (N = 14).

*Ab*, antibody; *ACC*, acute care clinic; *ED*, emergency department; *HCV*, hepatitis C virus; *VL*, viral load.
